# Real-Time Diagnostics on a QKD Link via QBER Time-Series Analysis

**DOI:** 10.3390/e26110922

**Published:** 2024-10-30

**Authors:** Georgios Maragkopoulos, Aikaterini Mandilara, Thomas Nikas, Dimitris Syvridis

**Affiliations:** 1 Department of Informatics and Telecommunications, National and Kapodistrian University of Athens, Panepistimiopolis, 15784 Ilisia, Greece; mandkat@di.uoa.gr (A.M.); tnikas@di.uoa.gr (T.N.); dsyvridi@di.uoa.gr (D.S.); 2 Eulambia Advanced Technologies, Agiou Ioannou 24, Building Complex C, 15342 Ag. Paraskevi, Greece

**Keywords:** QKD, ML

## Abstract

The integration of QKD systems in metro optical networks raises challenges that cannot be fully resolved with current technological means. In this work, we devised a methodology for identifying different types of impairments for a QKD link embedded in a communication network. Identification occurs in real time using a supervised machine learning model designed for this purpose. The model takes only QBER and SKR time-series data as the input, making its applicability not restricted to any specific QKD protocol or system. The output of the model specifies the working conditions for the QKD link, which is information that can be valuable for users and key management systems.

## 1. Introduction

After three decades of intense research [1] in quantum cryptographic protocols and their implementations, we are in the era where Quantum Key Distribution (QKD) systems are commercially available. While the technology in such devices is very advanced and keeps improving, there is a couple of important obstacles that prevent their generalized integration in existing metro optical networks. The first obstacle is the vulnerability of quantum signals to the effect of attenuation due to propagation in the fiber or due to the presence of network components. In QKD, quantum signals contain, on average, less than one photon, and therefore, a simple incident of a photon loss is destructive for the carried quantum information. Quantum repeaters [2], error-correcting codes [3], high-dimensional QKD protocols [4,5,6,7,8,9,10,11,12] and their related error correcting codes [13,14], and Twin-Field protocols [15] are designed to solve this problem, but the technology is still immature for their generalized application. The second important obstacle is the effect of photon addition to quantum signals co-propagating with classical ones in the same fiber. While numerous studies [16,17,18,19,20,21] were carried out on the performance of QKD systems under different conditions of coexistence with classical ones, the effect of photon addition still restricts, in the majority of cases, QKD signals to Single-mode Dark Fibers (SDFs).

In this work, we sought solutions offered by the field of machine learning (ML) for advancing the integration of QKD devices in classical communication networks to overcome the aforementioned obstacles. More specifically, we developed an ML model to classify different types of impairments that may occur in an active quantum channel, assuming access only to QBER and Secure Key Rate (SKR) data. The ML model serves as a substitute for a theoretical model of the system, which would be complicated to construct and specific to a particular QKD system. By training the parameters of the ML model using labeled data (based on the types of impairments), the model learns to respond to other unclassified data and impairments. The types of impairments we tested in this work included photon addition due to coexistence and photon loss due to attenuation. While it is generally understood that the presence of impairments on the transmission line decreases the SKR and increases the QBER, we demonstrated through our results that more information could be extracted from the time series of the QBER and SKR.

Our suggested methodology for analyzing the QBER/SKR time series follows the current trend of employing ML methods for improving the functionality of QKD [22,23,24,25,26,27,28,29,30,31,32], though its objective is distinct from other works. We constructed an ML model that can be trained to detect and classify impairments on a QKD link that is not isolated but embedded in a metro optical network. In this work, we demonstrated the developed methodology by emulating impairments caused by (a) coexisting classical signals in the C-band (with the quantum signal in the O-band [16,17,18]) and (b) fiber impairments leading to photon losses in the quantum signal. A network operator may choose to add other types of impairments to this list or further refine these two general classes of impairments, as we do in our demonstration. Since impairments directly influence the SKR and QBER, such a tool is crucial for the network operator and key management system to take prompt actions to address the causes of QKD performance degradation. For instance, if the model consistently detects photon loss impairments, the network operator can promptly use an OTDR to locate the source of the increased losses. Conversely, if coexistence is detected above a certain threshold, classical signals should be redirected within the network.

The structure of this manuscript is as follows. In Section 2, we present and justify the ML model that we constructed for extracting features from the QBER/SKR time series and, in succession, for performing the impairment classification. In order to test the validity of the suggested ML model, we collected training and test data under conditions that emulated different types of impairments. The experimental acquisition of data and the results of the impairment classification achieved by the ML model are reported in Section 3. Finally, in Section 4, we discuss perspectives regarding the method and future works.

## 2. The ML Model for Diagnosing the Status of a QKD Link

Our aim was to build an ML model that received input data from the QKD system and output the functioning class for the QKD link, chosen from a predetermined list of classes. QKD systems may differ in the quantum protocols implemented and the functions of the classical layer. However, every functioning QKD system generates time-series data for the SKR and QBER. To create an ML model that can be applied to any QKD system, we naturally selected SKR and QBER data as the input and treated them as time series to maximize the extracted information. To train the model, we first created labeled data by collecting the SKR and QBER data under normal operating conditions and various types of impairments. In what follows, we first explain the components of the ML model and then provide an example of collecting labeled data for training and prediction.

Time series are sequences of data points ordered in time, often analyzed for trends, seasonality, or anomalies. Some of the most widely used models in time series *prediction* include statistical models, such as ARIMA [33], or recurrent neural network models [34], such as LSTM. Our goal in this work was to train a model that is able to decide whether impairments on a QKD link exist and their types. This goal can be straightforwardly reduced to a classification task, which is not a prediction problem. In addition, the nature of QKD systems requires rapid classification using short sequences of recent data to ensure the timely detection of issues. Hence, in this work, we treated the QBER and SKR sequences as discrete batches of *N* data points. This batch-based approach allowed us to extract dynamic features from each batch of time-series data, a method that has shown to offer significant advantages [35] in classification tasks.

In this work, we extracted *k* features for each batch of *N* data points via Python’s (Version 3.10.14) package *tsfresh* [36], which provided various statistical, frequency-domain, and model-based characteristics from the raw time series. These included both simple statistical measures, such as the mean, variance, and quantiles, and more complex ones, such as ARIMA coefficients and Fourier and wavelet transformations. Typically, the extracted features are subsequently used as input for ML models, such as Neural Networks (NNs), which are trained to classify the batch of data into different categories. However, in our case, the *tsfresh* package extracted over k=1500 features for each batch of N=10 data points, and a feature space of such dimension is both computationally costly and prone to overfitting if treated by an NN. To tackle this issue, we applied an XGBoost model [37] to select the most important *K* features and then fed these into an NN. The XGBoost model contains free paramaters whose values are defined during the training phase. In Appendix A, we provide more information about this ML model. Let us note here that we could have used the selected features provided by the XGBoost model to perform the classification. However, as we explain in Section 3, complementing the pipeline with an NN improved the achieved accuracy of the classification without considerably increasing the computational time.

Therefore, the last element in the pipeline was an NN that performed the final classification task for the batch of time series data under study. The number of neurons in the input layer was naturally defined by the *K* number of features (provided by the XGBoost model), while the number of neurons in the output layer should match the number of classes. After the training phase, given an input, the (normalized) values of the neurons in the output layer provided the estimated probabilities for each class. There are different hyperparameters in an NN, e.g., number of layers, kind of activation function, and loss function, and one should perform an initial optimization on these. In the working example of Section 3, where we treated a problem with nine classes of impairments, and where we picked N=10 and K=50, we employed a deep NN that had a depth of three hidden layers: 50×128×256×128×9. There, we used ReLU activation functions with a cross-entropy loss and a 20-epoch training loop.

**Figure 1:**
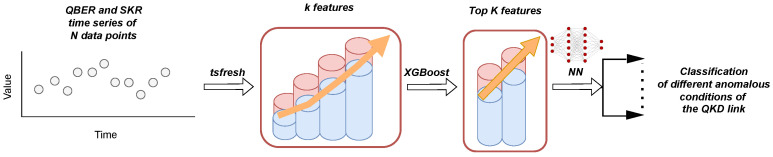
A schematic view of the ML pipeline used in this work for classifying different kind of impairments. While the QKD system is functioning and distributes keys, one acquires *N* sequential values of the QBER and SKR. *k* features are extracted from these time series with the *tsfresh* Python (Version 3.10.14) package, which, in sequence, are reduced to *K* features with the *XGBoost* method. The *K* values are fed in a deep NN for either training (during the training phase) or class prediction (during the prediction phase).

A schematic overview of the entire ML ‘pipeline’ is presented in Figure 1. The parameters inside the XGBoost and NN models are trained with QKD data under different distinct anomalous conditions for the QKD link to classify them. Then, by feeding the model with real-time QBER and SKR data, one can classify the current status of the QKD link accordingly.

Let us now describe with an example how the data acquisition in the training and prediction phases are implemented in real time, while also including the necessary step of data normalization.

**Training:**

- **Reference points and normalization:** Activate the QKD system and collect the first *N* data points. These data points are used as *reference* points, with all following time-series data being normalized according to the median values of the first using a MinMax scaler.
- **Creating labeled training data:** Proceed by acquiring sequential *N* QBER and SKR data while inducing a specific impairment on the transmission line of the quantum signal. The data are then labeled according to the type of impairment. Repeat for all other types of impairments.
- **Training the ML pipeline:** use all collected data to train the entire ML pipeline by minimizing the loss function.

**Prediction:**

- **Reference points and normalization:** A period of *N* time steps where the system runs without impairments is assumed. These *N* data points are used as a normalization reference for the forthcoming ones.
- **Classification in real-time:** at every time step, one may feed the batch created by the current point and N−1 previous ones into the ML pipeline in order to identify the impairments present in the QKD link.

It is important to note that the time frames for collecting data in each impairment mode vary between experiments, as the rate at which the QKD’s log file is updated correlates with the key rate and, consequently, with each impairment.

## 3. Demonstration of the Method with Experimental Data

The core of the experimental setting was a pair of Toshiba terminals, QKD4.2A-MU and QKD4.2B-MU (TOSHIBA DIGITAL SOLUTIONS CORPORATION, Kanagawa, Japan), that realized an advanced one-way phase-encoded protocol with coherent states, the so called T12 protocol [38]. The QKD Transmitter (Alice) sent quantum data at 1310 nm to the Receiver (Bob) via an SDF. In the same fiber, two low-power classical signals co-propagated at 1530 and 1529.30 nm. A second auxiliary fiber connected the terminals to establish classical communication from the Receiver to the Transmitter at 1528.77 nm. The power of all three classical channels was <+3 dBm.

**Figure 2:**
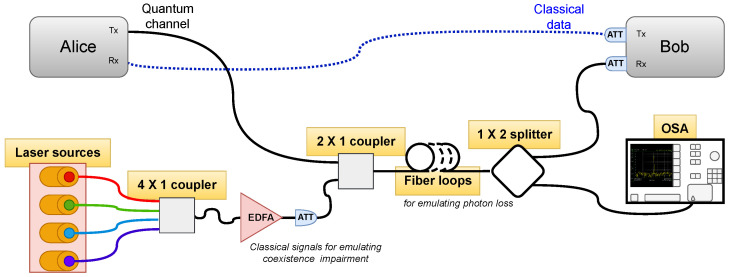
Experimental setting for the QBER/SKR data acquisition. From the bank of lasers, one could select the number (1–4) and power of the classical signals placed in the C-band coexisting with quantum data placed in the O-band. An Erbium-Doped Fiber Amplifier (EDFA) could be connected after the laser bank to further amplify the powers of the lasers. A number of fiber loops of diameter ≈2 cm could be formed along the QKD link to induce the photon losses. An Optical Spectrum Analyzer (OSA) (ANRITSU MS 9710c, ANRITSU, Morgan Hill, CA, USA) was used to monitor the attenuation in the fiber link.

The overall experimental setting for the training–test data acquisition under different conditions, which marked the different classes of impairments, is presented in Figure 2. One can use this setting to realize four different configurations for acquiring data: (a) normal mode, i.e., no impairments along the transmission line; (b) coexistence with optical cw signals without amplification via an EDFA; (c) coexistence with optical cw signals amplified by an EDFA; and (d) photon loss induced by small fiber loops. The length of the fiber for all experiments was kept at 2 km, while the attenuation along the transmission line due to the fiber and optical elements (couplers, attenuators) was −14 dB (as measured by an OSA) for all experiments. Below, we give quantitative details for the categorical classes of Table 1, namely, the wavelength λ and power *P* of the lasers (as measured by an OSA, see Figure 2), as well as the excess attenuation $A_{exc}$ due to the presence of the fiber loops. In the training phase of the coexistence with optical cw signals without an EDFA, we collected data for an interval of powers, as indicated below, and treated all these data under the same class/label. We performed this in order to investigate whether the model was capable of being trained for a wide class of events.

- **Class 1**—one laser: λ=1549.38 nm, $P=-\left\{23.5,21.7,20.5,19.55,18.84,18.37,18.1\right\}$ dBm.
- **Class 2**—two lasers: $\lambda_{1}=1549.38$, $\lambda_{2}=1549.46$ nm,
  $P_{1}=-\left\{23.5,21.7,20.5,19.55,18.84,18.37,18.1\right\}$,
  $P_{2}=-\left\{21.6,20.2,19.4,19.0,18.8,18.9,19.2\right\}$ dBm.
- Four lasers and EDFA: $\lambda_{1}=1548.5$, $\lambda_{2}=1549$, $\lambda_{3}=1549.5$, $\lambda_{4}=1550$ nm:
  **Class 3**—I=18 mA: $P_{1}=-17.9$, $P_{2}=-16.9$, $P_{3}=-15.6$, $P_{4}=-15.6$ dBm.
  **Class 4**—I=21 mA: $P_{1}=-16.5$, $P_{2}=-15.7$, $P_{3}=-14.6$, $P_{4}=-14.3$ dBm.
  **Class 5**—I=24 mA: $P_{1}=-15.5$, $P_{2}=-14.5$, $P_{3}=-13.4$, $P_{4}=-13.1$ dBm.
- **Class 6**—photon loss 20%: $A_{exc}=-0.9$ dB.
- **Class 7**—photon loss 46%: $A_{exc}=-1.9$ dB.
- **Class 8**—photon loss 67%: $A_{exc}=-3.1$ dB.

In the experiments, we made a distinction between the lasers with and without the EDFA since this type of amplifier incorporates an optical filter that decreases the outband ASE noise leaking to the O-band where the quantum channel resides. We did not register any Raman noise due to the short propagation length (2 km) of the classical (and quantum) signals [16,17,18]. Regarding the experiments where we simulated the effect of photon losses via the formation of small loops, in these formations, the radius of curvature of the fiber’s coating was decreased, impelling a part of the quantum light signal proportional to the number of loops to escape out of the fiber. This experiment emulated the action of an eavesdropper or the activation of an optical element, such as a coupler or multiplexer in the network, in a controllable way.

**Figure 3:**
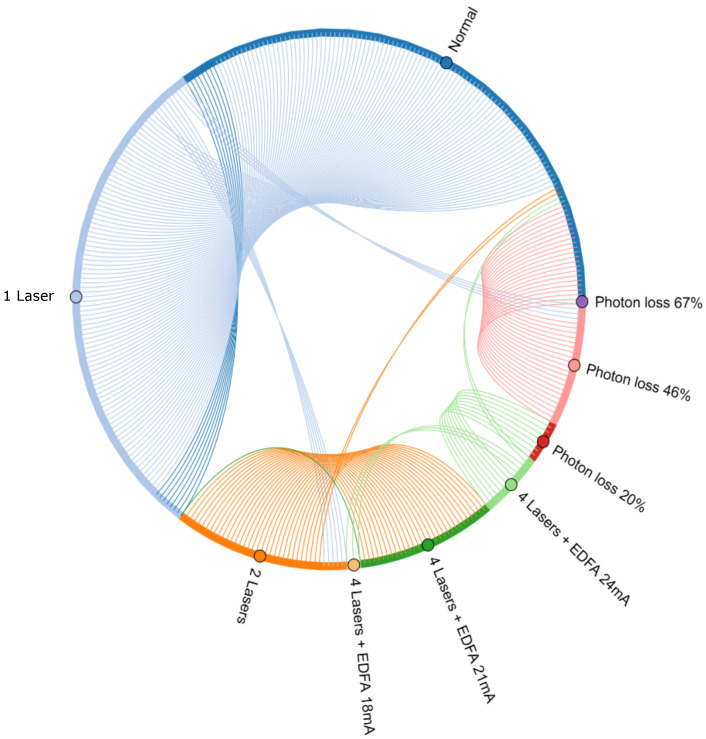
This chord diagram [39] visualizes the misclassification events for the test set. Each colored segment of the circle represents a class and the related arc length is proportional to the percentage of misclassified data for this class. The colored stripes indicate the misclassification of data from one class to another, having the color of the former. As an example, an orange stripe connects the orange class (2 lasers) to the dark green class (4 lasers + EDFA 21 mA). This stripe indicates that data points with the ground truth label ‘2 Lasers’ were misclassified as ‘4 Lasers + EDFA 21mA’.

We proceeded with the application of the ML methodology on the acquired data and we first split these as 80–20% for training–test, respectively. The test data were always derived from the tail end of the time series since a plain random split would result in a training set that contained data from time frames ahead of some test data, which could lead to misleading results. After training the XGBoost and NN models, predictions were made on the test set and the results are provided in Table 1. These are complemented by the confusion matrix in Table 2 and the chord diagram of Figure 3, which intends to give a visual representation of the misclassified data. The results in the table show that the *Precision*, i.e., the number of true positives divided by the total number of positive predictions, was high for all the impairment types. On the other hand, the classes ‘1 Laser’ and ‘2 Lasers’ were predicted less frequently than the others, as one can read from the *Recall* column. We correlated this outcome with the fact that for these specific classes, the data corresponding to the different levels of powers for the lasers were merged together. This argument is supported by the fact that for the cases where the EDFA was used, the model was able to distinguish between the different levels of power and the prediction outcomes were very good. Finally, the model showed the highest confidence in distinguishing the different levels of photon loss, particularly in the case of 67% photon loss, where the model made statistically no mistakes. The macro average of the *F1-score*, that is, the weighted average mean of the Precision and Recall, had a value of 0.89, and one may conclude that the developed methodology has the capacity to distinguish each class of simulated impairment with a high certainty.

It is worth noting that the XGBoost model alone, using all the features, achieved an average F1-score of 0.86, which was lower than the 0.89 achieved by the 50 features fed into the NN. In typical tabular data, an opposite trend is usually observed, leading us to conclude that for the given time series, the combination of XGBoost and NN was preferable. We also studied the performance of the NN with different numbers of K features and found that as K decreased, the performance also declined. Finally, it is important to emphasize that although the QBER and SKR data were theoretically correlated, we observed in practice that including both types of time series in the analysis significantly improved the classification results.

## 4. Conclusions

The events of photon loss and addition in quantum signals are limiting factors across all QKD implementations. In this work, we designed and successfully tested an ML methodology that, after the training phase, allowed for the real-time identification of these fundamental types of impairments. To the best of our knowledge, this is the first work dedicated to detecting anomalous conditions in a QKD link by analyzing information from a QBER time series.

In this study, the methodology was tested at a basic level by emulating anomalous conditions for the QKD link in the lab. We expect that applying this methodology in a QKD testbed will lead to further refinements in the parameters and structure of this initial model. Finally, an advantage of the developed methodology is its device-agnostic applicability, which we plan to demonstrate in future work.

## Figures and Tables

**Table 1:** Metrics that evaluated the classification results of the model. The medians and standard deviations (in parentheses) of the normalized QBER and SKR data are also listed for clarity.

Class #		Precision	Recall	F1-Score	QBER	SKR	# Data
**0**	Normal	0.90	1.00	0.95	−0.05 (0.18)	1.08 (0.16)	4064
**1**	1 laser	0.88	0.27	0.41	−0.03 (0.04)	1.04 (0.05)	395
**2**	2 lasers	0.93	0.77	0.85	0.28 (0.07)	0.85 (0.07)	466
**3**	4 lasers and EDFA (18 mA)	0.98	1.00	0.99	0.29 (0.03)	0.77 (0.04)	392
**4**	4 lasers and EDFA (21 mA)	0.96	0.99	0.98	0.3 (0.02)	0.83 (0.04)	2070
**5**	4 lasers and EDFA (24 mA)	1.00	0.87	0.93	0.17 (0.03)	0.87 (0.04)	353
**6**	Photon loss 20%	0.96	1.00	0.98	0.17 (0.03)	0.82 (0.04)	1067
**7**	Photon loss 46%	0.99	0.93	0.96	0.01 (0.03)	0.96 (0.06)	1529
**8**	Photon loss 67%	1.00	1.00	1.00	0.14 (0.03)	0.82 (0.04)	1629
	Accuracy			0.95			
	Macro average	0.96	0.87	0.89			

**Table 2:** The confusion matrix that reflects the misclassified events for the trained model.

Actual\Predicted	Class 0	Class 1	Class 2	Class 3	Class 4	Class 5	Class 6	Class 7	Class 8
**Class 0**	1049	8	0	0	0	0	0	0	0
**Class 1**	89	33	6	0	0	0	0	4	0
**Class 2**	2	0	79	0	30	0	0	0	0
**Class 3**	0	0	0	91	0	0	0	0	0
**Class 4**	0	0	1	0	607	0	0	0	0
**Class 5**	2	0	0	3	0	100	9	0	0
**Class 6**	0	0	0	0	0	0	270	0	0
**Class 7**	21	0	0	0	0	0	0	384	1
**Class 8**	0	0	0	0	0	0	0	0	414

## Data Availability

The experimental data and ML programs are available upon request to the corresponding author.

## Abbreviations

The following abbreviations are used in this manuscript:

QKD	Quantum Key Distribution
NN	Neural Network
SDF	Single-mode Dark Fibers
SKR	Secure Key Rate
ML	Machine learning
EDFA	Erbium-Doped Fiber Amplifier
OSA	Optical Spectrum Analyzer

## Appendix A

Here, we provide more information on the XGBoost model, which is habitually used for supervised learning tasks. This is a gradient-boosting algorithm that builds an ensemble of decision trees by sequentially training them to correct the errors of the previous trees. Its core process is called additive training, where new trees are added to the model until the error is minimized below a desired threshold.

The prediction ${\hat{y}}_{i}$ of the XGBoost for a data point $x_{i}$ is expressed as

(A1) $${\hat{y}}_{i}=\sum_{m=1}^{M}f_{m}\left(x_{i}\right)$$

where $f_{m}\left(x_{i}\right)$ is the prediction from the *m*-th decision tree, and *M* is the total number of decision trees. The objective function that the XGBoost minimizes is

(A2) $$L=\sum_{i}l\left(y_{i},{\hat{y}}_{i}\right)+\sum_{m}\Omega \left(f_{m}\right)$$

where $l(y_{i},{\hat{y}}_{i})$ is the chosen loss function, and $\Omega (f_{m})$ is a regularization term to control the complexity of each tree.

After the training, the XGBoost model additionally offers a measure of importance for each feature called the information gain. This measure quantifies how well a feature splits the data to reduce the uncertainty about the target class. Features that provide higher information gain contribute more effectively to distinguishing between different classes, which, in our case, were the impairments. It measures the reduction in entropy when splitting on a particular feature $X_{j}$. The information gain for a particular feature $X_{j}$ is given by

(A3) $$IG\left(X_{j}\right)=H\left(Y\right)-H\left(Y|X_{j}\right)$$

where H(Y) is the entropy of the target variable *Y*, representing the uncertainty in the class labels, and $H(Y|X_{j})$ is the conditional entropy of *Y* given the feature $X_{j}$. Then, the importance of each feature is ordered by the information gain, and the top *K* features are selected as inputs for the NN.
